# Supplementary material for: 12-Oxophytodienoate Reductase Overexpression Compromises Tolerance to Botrytis cinerea in Hexaploid and Tetraploid Wheat
Source: Plants (Basel). 2023 May 22;12(10):2050. doi: 10.3390/plants12102050 (PMC10222670; doi:10.3390/plants12102050)
Supplement: Supplementary file 1 [file plants-12-02050-s001.zip › plants-2387127-supplementary.pdf]

Supplementary Table S1. Segregation analysis of *GFP* expression in T1 progeny of T0 transgenic plants of tetraploid emmer wheat 'Runo'

T0 plant	Number of T1 seeds tested	Seedlings T1			
		Observed *GFP* segregation ratio (positive:negative)	Ratio	χ^2 value for the expected segregation ratio (positive:negative)	
				3:1	15:1
RC-1	150	147:3	49.0:1	42.32	4.62
RC-2b	99	79:20	4.0:1	1.22	32.89
RC-3	89	56:33	1.7:1	6.93	144.36
RC-7	104	65:39	1.7:1	8.67	173.33
RC-10	74	46:28	1.6:1	6.50	126.01
RC-12	87	64:23	2.8:1	0.10	60.51
RC-13	99	49:50	1.0:1	34.35	330.91
RC-16	112	98:14	7.0:1	9.33	7.47
RC-17	111	73:38	1.9:1	5.05	148.35
RC-23a	76	53:23	2.3:1	1.12	74.79
RC-26	48	31:11	3.4:1	0.11	22.76
RC-28	133	42:91	0.5:1	133.74	877.36
RC-29	54	37:17	2.2:1	1.21	58.67
RC-30	128	87:41	2.1:1	3.38	145.20
RC-33	109	81:28	2.9:1	0.03	70.29

if χ^2 value is below 3.84 ($P > 0.05$) the observed segregation ratio is not significantly different from the expected ratio

Supplementary Table S2. List of primers used in this study

Primer name	Sequence (5' – 3')	Product length, bp	Purpose
AtOPR3mid	CCGAAGGCACCATGGTCTCTCCC<br>TCGGAAGCTTCTAAAGCCCGAGG	295	RT-PCR
AtOPR3a	ACGTGCTTCTCATGCAGTGT<br>ACTTCACGTGGGAACCATCG	116	qPCR
GFP	GCGACGTAAACGGCCACAAG<br>CCAGCAGGACCATGTGTGATCG	606	RT-PCR
TaWIN	TTTTCTGTGTTCTACTATGAGATCTTGAA<br>AAGTGCATAATTAAACAGAGGTAGTGATG	348	RT-PCR<br>qPCR

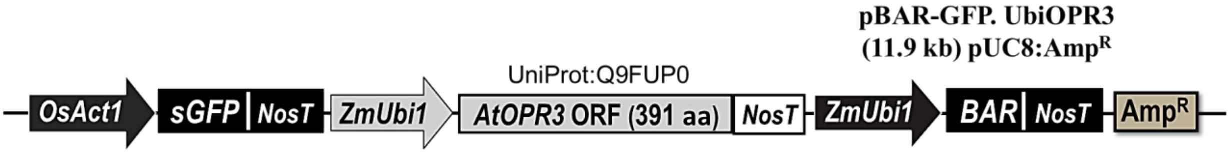

Figure S1: Schematic representation of the pBAR-GFP.UbiOPR3 expression cassette used for emmer wheat transformation. *OsAct1*, rice *Actin 1* promoter; *ZmUbi1*, maize *Ubiquitin 1* promoter; *NosT*, *Nopaline Synthase* terminator; *sGFP*, modified (S65T) *Green Fluorescent Protein* gene; *BAR*, BASTA resistance gene (phosphinothricin acetyl transferase); *AmpR*, ampicillin resistance gene.

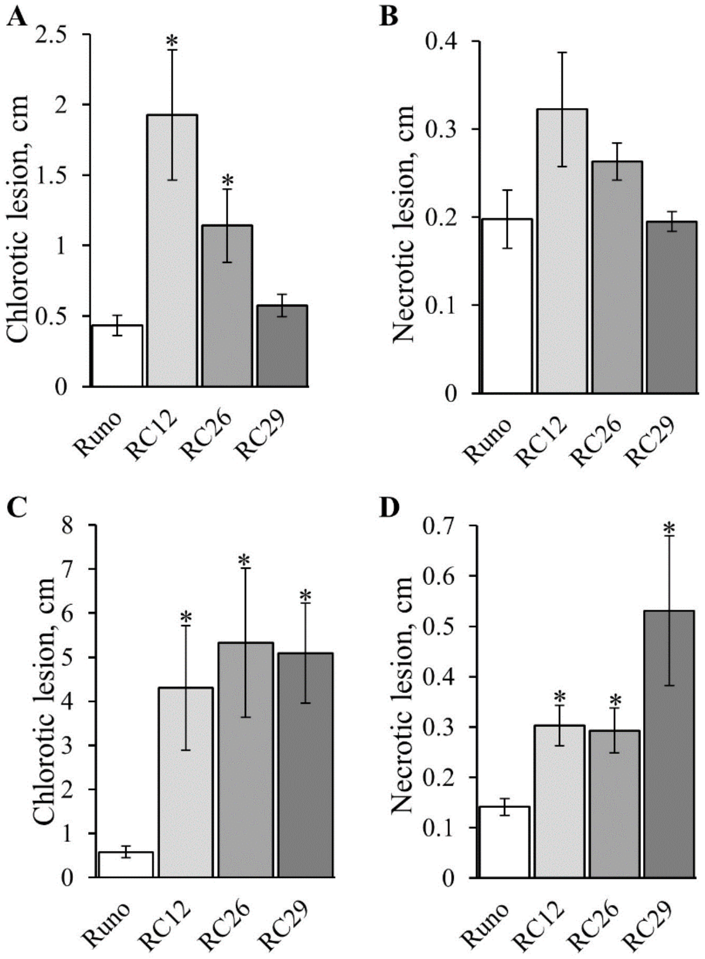

Figure S2. The tolerance of transgenic tetraploid wheat plants to *B. cinerea*. The size of the chlorotic lesions (a and c) and necrotic lesions (b and d) formed on the second (a and b) and third (c and d) leaves infected with *B. cinerea* on the fifth day after infection. Data are averages of at least three biological replicas consisting of 4-5 plants ± SE. Stars indicate a statistically significant difference between transgenic lines and non-transgenic control at $p \leq 0.05$ assessed by the Student's t test.

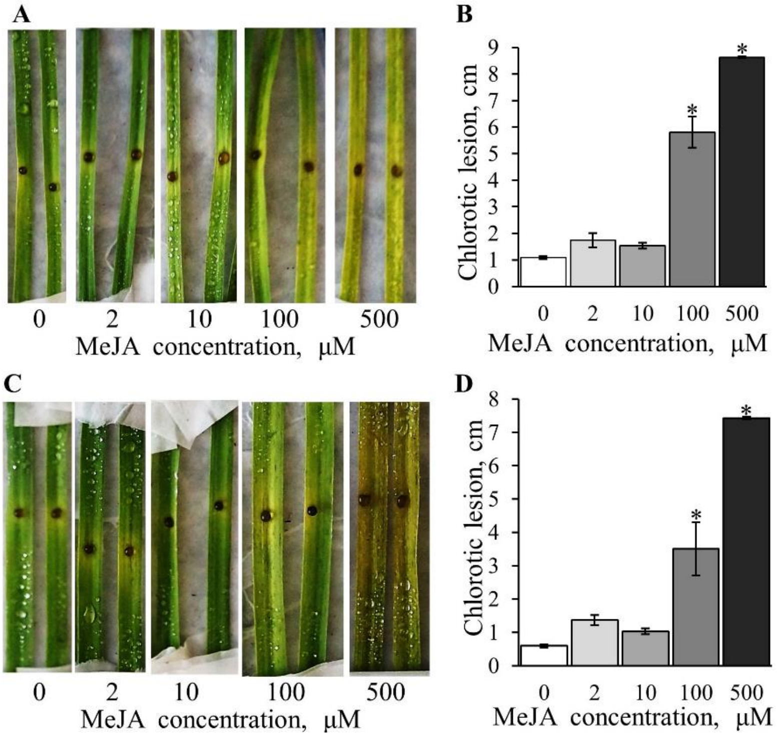

Figure S3. Effect of MeJA on *B. cinerea* infection development on the detached first (a and b) and third (c and d) leaves of hexaploid wheat on the 3rd day after infection. A and c, are representative images of infected leaves; b and d – size of the lesion zone. Stars indicate a statistically significant difference from the non-treated with MeJA control at p ≤ 0.05 assessed by the Student's t test.

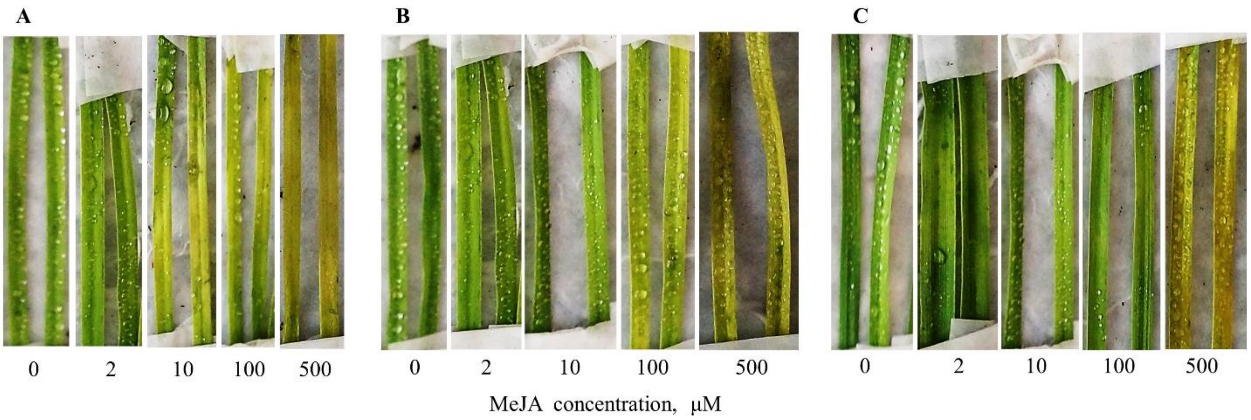

Figure S4. Effect of MeJA on the Sar-60 leaf yellowing, 1st (a), 2nd (b), and 3rd (c) leaves.
